# Evolution of the most common English words and phrases over the centuries

**DOI:** 10.1098/rsif.2012.0491

**Published:** 2012-07-25

**Authors:** Matjaž Perc

**Affiliations:** Faculty of Natural Sciences and Mathematics, University of Maribor, Koroška cesta 160, 2000 Maribor, Slovenia

**Keywords:** Zipf's law, preferential attachment, English language

## Abstract

By determining the most common English words and phrases since the beginning of the sixteenth century, we obtain a unique large-scale view of the evolution of written text. We find that the most common words and phrases in any given year had a much shorter popularity lifespan in the sixteenth century than they had in the twentieth century. By measuring how their usage propagated across the years, we show that for the past two centuries, the process has been governed by linear preferential attachment. Along with the steady growth of the English lexicon, this provides an empirical explanation for the ubiquity of Zipf's law in language statistics and confirms that writing, although undoubtedly an expression of art and skill, is not immune to the same influences of self-organization that are known to regulate processes as diverse as the making of new friends and World Wide Web growth.

## 1. Introduction

The evolution of language [1–7] is, much like the evolution of cooperation [8,9], something that markedly distinguishes humans from other species [10,11]. While the successful evolution of cooperation enables us to harvest the benefits of collective efforts on an unprecedented scale, the evolution of language, along with the set of grammatical rules [12] that allows infinitely many comprehensible formulations [13–16], enables us to uphold a cumulative culture [17]. Were it not for books, periodicals and other publications, we would hardly be able to continuously elaborate over what is handed over by previous generations, and, consequently, the diversity and efficiency of our products would be much lower than it is today. Indeed, it seems like the importance of the written word for where we stand today as a species cannot be overstated.

The availability of vast amounts of digitized data, also referred to as ‘metaknowledge’ or ‘big data’ [18], along with the recent advances in the theory and modelling of social systems in the broadest possible sense [19,20], enables quantitative explorations of the human culture that were unimaginable even a decade ago. From human mobility patterns [21,22], crashes in financial markets [23] and in our economic life [24,25], the spread of infectious diseases [26–28] and malware [29,30], the dynamics of online popularity [31] and social movements [32], to scientific correspondence [33,34], there appear to be no limits to insightful explorations that lift the veil on how we as humans behave, interact, communicate and shape our very existence.

Much of what we have learned from these studies strongly supports the fact that universal laws of organization govern how nature, as well as we as a society, work [35,36]. Languages, as comprehensively reviewed by Solé *et al.* [37], and as suggested already by Zipf [38] as well as by others before him [39], are certainly no exception. In fact, in many ways, it seems more like it is the other way around. Zipf's law is frequently related to the occurrence of power-law distributions in empirical data [40], with examples ranging from income rankings and population counts of cities to avalanche and forest-fire sizes [41]. Yet the mechanisms that may lead to the emergence of scaling in various systems differ. The proposal made by Zipf was that there is tension between the efforts of the speaker and the listener, and it has been shown that this may indeed explain the origins of scaling in the human language [42]. The model proposed by Yule [43], relying on the rich-get-richer phenomenon (see [44] for a review), is also frequently cited as the reason for the emergence of Zipf's law. With the advent of contemporary network science [45–47], however, growth and preferential attachment, used ingeniously by Barabási & Albert [46] to explain the emergence of scaling in random networks, has received overwhelming attention, also in relation to the emergence of Zipf's law in different corpora of the natural language [48,49].

Here we make use of the data that accompanied the seminal study by Michel *et al.* [50], and show empirically, based on a large-scale statistical analysis of the evolution of the usage of the most common words and phrases in the corpus of the English books over the past five centuries, that growth and preferential attachment played a central role in determining the longevity of popularity and the emergence of scaling in the examined corpus. The presented results support previous theoretical studies [37] and indicate that writing, on a large scale, is subject to the same fundamental laws of organization that determine so many other aspects of our existence.

## 2. Results

**Figure 1.**
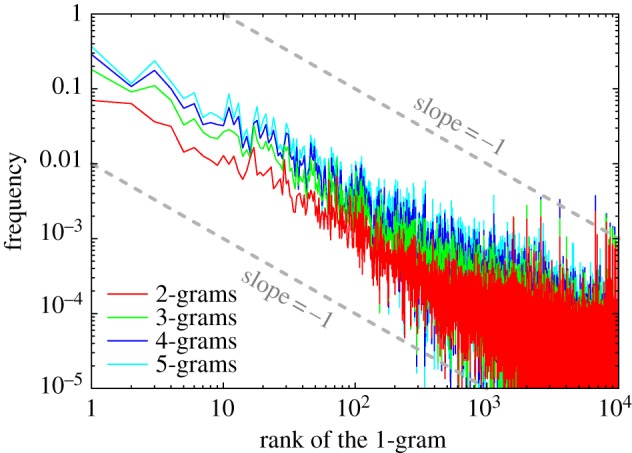
Confirmation of Zipf's law in the examined corpus. By measuring the frequency of 1-grams in the *n*-grams, where *n* > 2 (refer to key), we find that it is inversely proportional to the rank of the 1-grams. For all *n*, the depicted curves decay with a slope of −1 on a double log scale over several orders of magnitude, thus confirming the validity of Zipf's law in the examined dataset.

Henceforth we will, for practical reasons, refer to the words and phrases as *n*-grams [50], with the meaning as described in appendix A. We begin with presenting the results of a direct test of Zipf's law for the overall most common 1-grams in the English corpus since the beginning of the sixteenth century. For this purpose, we treat the *n*-grams for different *n* > 1 as individual corpora where the frequencies of the 1-grams are to be determined. Results presented in figure 1 confirm that, irrespective of *n*, the frequency of any given 1-gram is roughly inversely proportional to its rank. The ragged outlay of the curves is a consequence of the rather special construction of the corpora on which this test was performed. Yet, given the time span and the extent of the data, this is surely a very satisfiable outcome of a test for a century-old law [39,51] on such a large scale, validating the dataset against the hallmark statistical property of the human language.

**Figure 2.**
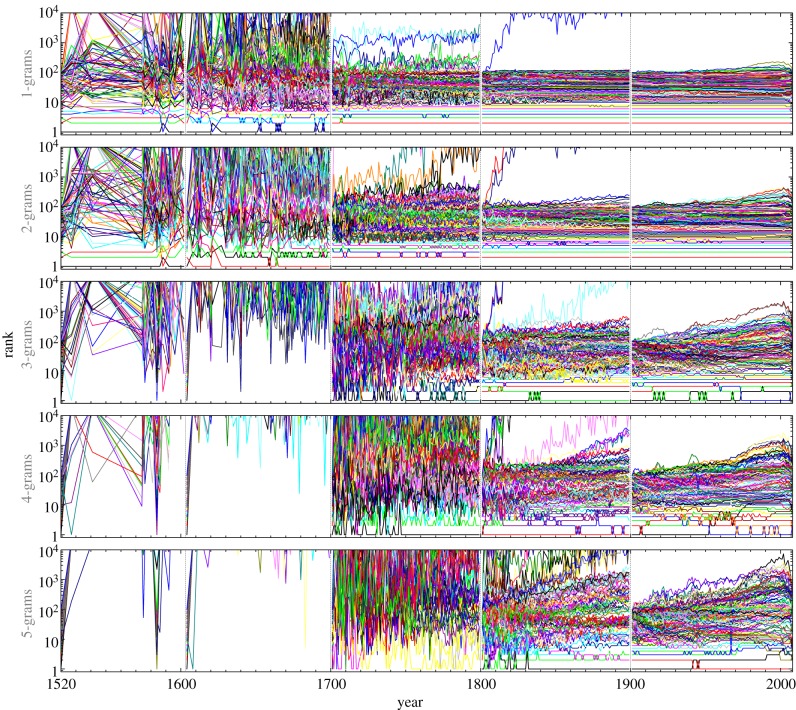
Evolution of popularity of the top 100 *n*-grams over the past five centuries. For each of the 5 starting years, being 1520, 1600, 1700, 1800 and 1900 from left to right (separated by dashed grey lines), the rank of the top 100 *n*-grams was followed until it exceeded 10 000 or until the end of the century. From top to bottom, the panels depict results for different *n*, as indicated vertically. The advent of the nineteenth century marks a turning point after which the rankings began to gain markedly on consistency. Regardless of which century is considered, the higher the *n* the more fleeting the popularity. Tables listing the top *n*-grams for all available years are available at http://www.matjazperc.com/ngrams.

Turning to the evolution of popularity, we show in figure 2 how the rank of the top 100 *n*-grams, as determined in the years 1520, 1604, 1700, 1800 and 1900, varied until the beginning of the next century. During the sixteenth and the seventeenth centuries, popularity was very fleeting. Phrases that were used most frequently in 1520, for example, only intermittently succeeded in re-entering the charts in the later years, despite the fact that we have kept track of the top 10 000 *n*-grams and have started with the top 100 *n*-grams in each of the considering starting years. It was not before the end of the eighteenth century that the top 100 *n*-grams gradually began succeeding in transferring their start-up ranks over to the next century. The longevity and persistency of popularity is the highest during the twentieth century, which is also the last one for which data are available, apart from the 8 years into the twenty-first century. Comparing the different *n*-grams with one another, we find that the 1-grams were always, regardless of the century considered, more likely to retain their top rankings than the 3-grams, which in turn outperformed the 5-grams. This, however, is an expected result, given that single words and short phrases are obviously more likely to be reused than phrases consisting of three, four or even, five words.

**Figure 3.**
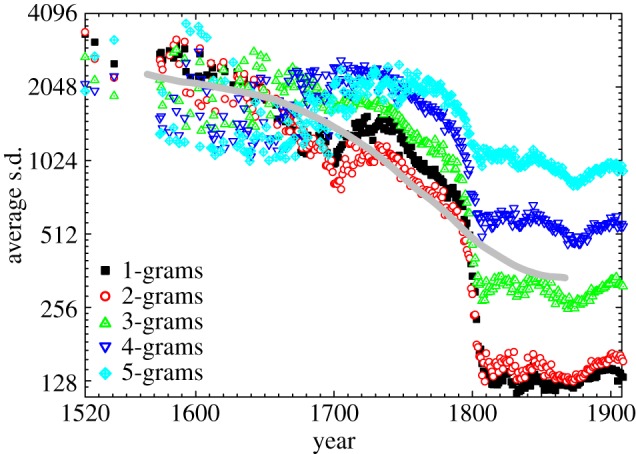
‘Statistical’ coming of age of the English language. Symbols depict results for different *n* (refer to key), as obtained by calculating the average standard deviation of the rank for the top 1000 *n*-grams 100 years into the future. The thick grey line is a moving average over all the *n*-grams and over the analysis going 50 and 100 years into the future as well as backwards. There is a sharp transition to a greater maturity of the rankings taking place at around the year 1800. Although the moving average softens the transition, it confirm that the ‘statistical’ coming of age was taking place and that the nineteenth century was crucial in this respect.

Although the fleeting nature of the top rankings recorded in the sixteenth and the seventeenth centuries is, to a degree, surely a consequence of the relatively sparse data (only a few books per year) if compared with the nineteenth and the twentieth centuries, it nevertheless appears intriguing as it is based on the relative yearly usage frequencies of the *n*-grams. Thus, at least a ‘statistical’ coming of age of the written word imposes as a viable interpretation. To quantify it accurately, we have conducted the same analysis as presented in figure 2 for the top 1000 *n*-grams for all years with data, and subsequently calculating the average standard deviation of the resulting 1000 curves for each starting year. Symbols presented in figure 3 depict the results of this analysis separately for all the *n*-grams. A sharp transition towards a higher consistency of the rankings occurs at the brink of the nineteenth century for all *n*, thus giving results presented in figure 2 a more accurate quantitative frame. These results remain valid if the rankings are traced only 50 years into the future, as well as if performing the same analysis backwards in time, as evidenced by the thick grey line depicting a moving average over this four scenarios as well as over all the *n*.

**Figure 4.**
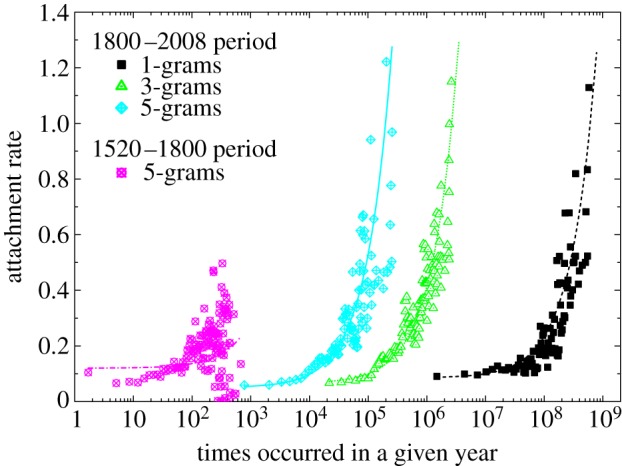
Emergence of linear preferential attachment during the past two centuries. Based on the preceding evolution of popularity, two time periods were considered separately, as indicated in the figure legend. While preferential attachment appears to have been in place already during the 1520–1800 period, large deviations from the linear dependence (the goodness-of-fit is ≈0.05) hint towards inconsistencies that may have resulted in heavily fluctuated rankings. The same analysis for the nineteenth and the twentieth centuries provides much more conclusive results. For all *n* the data fall nicely onto straight lines (the goodness-of-fit is ≈0.8), thus indicating that continuous growth and linear preferential attachment have shaped the large-scale organization of the writing of English books over the past two centuries. Results for those *n*-grams that are not depicted are qualitatively identical for both periods of time.

Both the validity of Zipf's law across all the data considered in this study, as well as the peculiar evolution of popularity of the most frequently used *n*-grams over the past five centuries, hint towards large-scale organization gradually emerging in the writing of the English books. Since the groundbreaking work by Barabási and Albert on the emergence of scaling in random networks [46], growth and preferential attachment has become synonymous for the emergence of power laws and leadership in complex systems. Here we adopt this beautiful perspective and test whether it holds true also for the number of occurrences of the most common words and phrases in the English books that were published in the past five centuries. In the seminal paper introducing culturomics [50], it was pointed out that the size of the English lexicon has grown by 33 per cent during the twentieth century alone. As for preferential attachment, we present in figure 4 evidence indicating that the higher the number of occurrences of any given *n*-gram, the higher the probability that it will occur even more frequently in the future. More precisely, for the past two centuries, the points quantifying the attachment rate follow a linear dependence, thus confirming that both growth and linear preferential attachment are indeed the two processes governing the large-scale organization of writing. Performing the same analysis for the preceding three centuries fails to deliver the same conclusion, although the seed for what will eventually emerge as linear preferential attachment is clearly inferable.

## 3. Discussion

The question ‘Which are the most common words and phrases of the English language?’ alone has a certain appeal, especially if one is able to use digitized data from millions of books dating as far back as the early sixteenth century [50] to answer it. On the other hand, writing about the evolution of a language without considering grammar or syntax [13], or even without being sure that all the considered words and phrases actually have a meaning, may appear prohibitive to many outside the physics community. Yet, it is precisely this detachment from detail and the sheer scale of the analysis that enables the observation of universal laws that govern the large-scale organization of the written word. This does not mean that the presented results are no longer valid if we made sure to analyse only words and phrases that actually have meaning or if we had distinguished between capitalized words, but rather that such details do not play a decisive role in our analysis. Regardless of whether a word is an adjective or a noun, or whether it is currently trendy or not, with the years passing by the mechanism of preferential attachment will make sure that the word will obtain its rightful place in the overall rankings. Together with the continuous growth of the English lexicon, we have a blueprint for the emergence of Zipf's law that is derived from a vast amount of empirical data and supported by theory [46]. This does not diminish the relevance of the tension between the efforts of the speaker and the listener [42], but adds to the importance of the analysis of ‘big data’ with methods of statistical physics [52,53] and network science [48,49,54] for our understanding of the large-scale dynamics of human language.

The allure of universal laws that might describe the workings of our society is large [35]. Observing Zipf's law [38], or more generally a power-law distribution [41], in a dataset is an indication that some form of large-scale self-organization might be taking place in the examined system. Implying that initial advantages are often self-amplifying and tend to snowball over time, preferential attachment, known also as the rich-get-richer phenomenon [43], the ‘Matthew effect’ [55], or the cumulative advantage [56], has been confirmed empirically by the accumulation of citations [57] and scientific collaborators [58,59], by the growth of the World Wide Web [36], and by the longevity of one's career [60]. Examples based solely on theoretical arguments, however, are many more and much easier to come by. Empirical validations of preferential attachment require large amounts of data with time stamps included. It is the increasing availability of such datasets that appears to fuel progress in fields ranging from cell biology to software design [61], and as this study shows, it helps reveal why the overall rankings of the most common English words and phrases are unlikely to change in the near future, as well as why Zipf's law emerges in written text.

## Appendix A. Methods

A.1. Raw data

The seminal study by Michel *et al.* [50] was accompanied by the release of a vast amount of data composed of metrics derived from approximately 4 per cent of books ever published. Raw data, along with usage instructions, are available at http://books.google.com/ngrams/datasets as counts of *n*-grams that appeared in a given corpus of books published in each year. An *n*-gram is made up of a series of *n* 1-grams, and a 1-gram is a string of characters uninterrupted by a space. Although we have excluded 1-grams that are obviously not words (for example, if containing characters outside the range of the ASCII table) from the analysis, some (mostly typos) might have nevertheless found their way into the top rankings. The latter were composed by recursively scanning all the files from the English corpus associated with a given *n* in the search for those *n*-grams that had the highest usage frequencies in any given year. Tables listing the top 100, top 1000 and top 10 000 *n*-grams for all available years since 1520 inclusive, along with their yearly usage frequencies and direct links to the Google Books Ngram Viewer, are available at http://www.matjazperc.com/ngrams.

A.2. Zipf's law

Taking the top 10 000 *n*-grams for all available years as the basis, we have determined the number of unique *n*-grams through the centuries and ranked them according to the total number of occurrences in the whole corpus during all the years. In this way, we have obtained a list of 148 557 unique 1-grams, 291 661 unique 2-grams, 482 503 unique 3-grams, 742 636 unique 4-grams and 979 225 unique 5-grams. This dataset was used for testing Zipf's law by searching for the overall top ranked 1-grams in all the other *n*-grams (*n* > 1) and recording their frequency of occurrence. For example, the 1-gram ‘the’ appeared in 22 826 of the 291 661 2-grams, hence its frequency is approximately 7.8 per cent. By plotting the so obtained frequency in dependence on the rank of the 1-grams for *n* = 2, 3, 4, 5 on a double log scale (figure 1), we observe four inversely proportional curves, thus confirming Zipf's law in the constructed dataset.

A.3. Attachment rate

Based on the assumption that the more frequently a given *n*-gram appears, the more linked it is to other *n*-grams, we have determined the attachment rate following network science [58] as follows. If an *n*-gram has appeared *m* times in the year *y*, and *k* times in the year *y* + Δ*y*, the attachment rate is *α*(*m*) = *k/m*Δ*y*. Note that the occurrences in the dataset are not cumulative. Hence there is no difference between *k* and *m* in the numerator. Moreover, by the determination of the attachment rate, we are not interested in the relative yearly usage frequencies, but rather in the absolute number of times a given *n*-gram has appeared in the corpus in any given year. Thus, *m* and *k* are not normalized with the total word counts per year. We have determined *α*(*m*) based on the propagation of top 100 *n*-grams between 1520–1800 and 1800–2008 with a yearly resolution. Missing years were bridged by adjusting Δ*y* accordingly. For the final display of the attachment rate in figure 4 and the linear fitting, we have averaged *α*(*m*) over approximately 200 non-overlapping segments in *m*.

## References

[1] Nowak M. A. Krakauer D. 1999 The evolution of language. Proc. Natl Acad. Sci. USA 96, 8028–8033 10.1073/pnas.96.14.8028 (doi:10.1073/pnas.96.14.8028) 10393942 PMC22182

[2] Hauser M. D. Chomsky N. Fitch W. T. 2002 The faculty of language: what is it, who has it, and how did it evolve? Science 298, 1569–1579 10.1126/science.298.5598.1569 (doi:10.1126/science.298.5598.1569) 12446899

[3] Nowak M. A. Komarova N. L. Niyogi P. 2002 Computational and evolutionary aspects of language. Nature 417, 611–617 10.1038/nature00771 (doi:10.1038/nature00771) 12050656

[4] Abrams D. Strogatz S. H. 2003 Modelling the dynamics of language death. Nature 424, 900 10.1038/424900a (doi:10.1038/424900a) 12931177

[5] Solé R. V. 2005 Syntax for free? *Nature* 434, 289 10.1038/434289a (doi:10.1038/434289a) 15772637

[6] Lieberman E. Michel J. B. Jackson J. Tang T. Nowak M. A. 2007 Quantifying the evolutionary dynamics of language. Nature 713–716 10.1038/nature06137 (doi:10.1038/nature06137) 17928859 PMC2460562

[7] Loreto V. Steels L. 2007 Social dynamics: emergence of language. Nature Physics 3, 758–760 10.1038/nphys770 (doi:10.1038/nphys770)

[8] Sigmund K. 2010 The calculus of selfishness. Princeton, MA: Princeton University Press

[9] Nowak M. A. Highfield R. 2011 SuperCooperators: altruism, evolution, and why we need each other to succeed. New York, NY: Free Press

[10] Miller G. 1981 Language and speech. San Francisco, CA: Freeman

[11] Hrdy S. B. 2011 Mothers and others: the evolutionary origins of mutual understanding. Cambridge, MA: Harvard University Press

[12] Nowak M. A. Komarova N. L. Niyogi P. 2001 Evolution of universal grammar. Science 291, 114–118 10.1126/science.291.5501.114 (doi:10.1126/science.291.5501.114) 11141560

[13] Chomsky N. 1965 Aspects of the theory of syntax. Cambridge, MA: MIT Press

[14] Hauser M. D. 1996 The evolution of communication. Cambridge, MA: MIT Press

[15] Lightfoot D. 1999 The development of language: acquisition, change and evolution. Oxford, UK: Blackwell

[16] Niyogi P. 2006 The computational nature of language learning and evolution. Cambridge, MA: MIT Press

[17] Lehman H. C. 1947 The exponential increase in man's cultural output. Social Forces 25, 281–290 10.1093/sf/25.3.281 (doi:10.1093/sf/25.3.281)

[18] Evans J. A. Foster J. G. 2011 Metaknowledge. Science 331, 721–725 10.1126/science.1201765 (doi:10.1126/science.1201765) 21311014

[19] Lazer D. 2009 Computational social science. Science 323, 721–723 10.1126/science.1167742 (doi:10.1126/science.1167742) 19197046 PMC2745217

[20] Castellano C. Fortunato S. Loreto V. 2009 Statistical physics of social dynamics. Rev. Mod. Phys. 81, 591–646 10.1103/RevModPhys.81.591 (doi:10.1103/RevModPhys.81.591)

[21] González M. C. Hidalgo C. A. Barabási A. L. 2008 Understanding individual human mobility patterns. Nature 453, 779–782 10.1038/nature06958 (doi:10.1038/nature06958) 18528393

[22] Song C. Qu Z. Blumm N. Barabási A. L. 2010 Limits of predictability in human mobility. Science 327, 1018–1021 10.1126/science.1177170 (doi:10.1126/science.1177170) 20167789

[23] Preis T. Stanley H. E. 2011 Bubble trouble: can a law describe bubbles and crashes in financial markets? Phys. World 24, 29–32

[24] Preis T. Reith D. Stanley H. E. 2010 Complex dynamics of our economic life on different scales: insights from search engine query data. Phil. Trans. R. Soc. A 368, 5707–5719 10.1098/rsta.2010.0284 (doi:10.1098/rsta.2010.0284) 21078644

[25] Preis T. Moat H. S. Stanley H. E. Bishop S. R. 2012 Quantifying the advantage of looking forward. Sci. Rep. 2, 350 10.1038/srep00350 (doi:10.1038/srep00350) 22482034 PMC3320057

[26] Liljeros F. Edling C. R. Amaral L. A. N. 2003 Sexual networks: implications for the transmission of sexually transmitted infections. Microbes Infect. 5, 189–196 10.1016/S1286-4579(02)00058-8 (doi:10.1016/S1286-4579(02)00058-8) 12650777

[27] Balcan D. Colizza V. Gonçalves B. Hu H. Ramasco J. J. Vespignani A. 2009 Multiscale mobility networks and the spatial spreading of infectious diseases. Proc. Natl Acad. Sci. USA 106, 21 484–21 489 10.1073/pnas.0906910106 (doi:10.1073/pnas.0906910106) PMC2793313 20018697

[28] Meloni S. Arenas A. Moreno Y. 2009 Traffic-driven epidemic spreading in finite-size scale-free networks. Proc. Natl Acad. Sci. USA 106, 16 897–16 902 10.1073/pnas.0907121106 (doi:10.1073/pnas.0907121106) PMC2761312 19805184

[29] Hu H. Myers S. Colizza V. Vespignani A. 2009 Wifi networks and malware epidemiology. Proc. Natl Acad. Sci. USA 106, 1318–1323 10.1073/pnas.0811973106 (doi:10.1073/pnas.0811973106) 19171909 PMC2635807

[30] Wang P. González M. Hidalgo C. A. Barabási A. L. 2009 Understanding the spreading patterns of mobile phone viruses. Science 324, 1071–1076 10.1126/science.1167053 (doi:10.1126/science.1167053) 19342553

[31] Ratkiewicz J. Fortunato S. Flammini A. Menczer F. Vespignani A. 2010 Characterizing and modeling the dynamics of online popularity. Phys. Rev. Lett. 105, 158701 10.1103/PhysRevLett.105.158701 (doi:10.1103/PhysRevLett.105.158701) 21230945

[32] Borge-Holthoefer J. 2011 Structural and dynamical patterns on online social networks: the Spanish May 15th movement as a case study. PLoS ONE 6, e23883. 10.1371/journal.pone.0023883 (doi:10.1371/journal.pone.0023883) PMC3158778 21886834

[33] Barabási A. L. 2005 The origin of bursts and heavy tails in humans dynamics. Nature 435, 207–211 10.1038/nature03459 (doi:10.1038/nature03459) 15889093

[34] Malmgren R. D. Stouffer D. B. Campanharo A. S. L. O. Amaral L. A. N. 2009 On universality in human correspondence activity. Science 325, 1696–1700 10.1126/science.1174562 (doi:10.1126/science.1174562) 19779200

[35] Bak P. 1996 How nature works: the science of self-organised criticality. New York, NY: Copernicus Press

[36] Newman M. E. J. Barabási A. L. Watts D. J. 2006 The structure and dynamics of networks. Princeton, NJ: Princeton University Press

[37] Solé R. V. Corominas-Murtra B. Fortuny J. 2010 Diversity, competition, extinction: the ecophysics of language change. J. R. Soc. Interface 7, 1647–1664 10.1098/rsif.2010.0110 (doi:10.1098/rsif.2010.0110) 20591847 PMC2988263

[38] Zipf G. K. 1949 Human behavior and the principle of least-effort. Reading, MA: Addison-Wesley

[39] Manning C. D. Schütze H. 1999 Foundations of statistical natural language processing. Cambridge, MA: MIT Press

[40] Clauset A. Shalizi C. R. Newman M. E. J. 2009 Power-law distributions in empirical data. SIAM Rev. 51, 661–703 10.1137/070710111 (doi:10.1137/070710111)

[41] Newman M. E. J. 2005 Power laws, pareto distributions and Zipf's law. Contemp. Phys. 46, 323–351 10.1080/00107510500052444 (doi:10.1080/00107510500052444)

[42] Ferrer i Cancho R. Solé R. V. 2003 Least effort and the origins of scaling in human language. Proc. Natl Acad. Sci. USA 100, 788–791 10.1073/pnas.0335980100 (doi:10.1073/pnas.0335980100) 12540826 PMC298679

[43] Yule G. U. 1925 A mathematical theory of evolution, based on the conclusions of Dr J. C. Willis, F.R.S. Phil. Trans. R. Soc. Lond. B 213, 21–85 10.1098/rstb.1925.0002 (doi:10.1098/rstb.1925.0002)

[44] Simkin M. V. Roychowdhury V. P. 2011 Re-inventing willis. Phys. Rep. 502, 1–35 10.1016/j.physrep.2010.12.004 (doi:10.1016/j.physrep.2010.12.004)

[45] Watts D. J. Strogatz S. H. 1998 Collective dynamics of ‘small-world’ networks. Nature 393, 440–442 10.1038/30918 (doi:10.1038/30918) 9623998

[46] Barabási A.-L. Albert R. 1999 Emergence of scaling in random networks. Science 286, 509–512 10.1126/science.286.5439.509 (doi:10.1126/science.286.5439.509) 10521342

[47] Albert R. Barabási A. L. 2002 Statistical mechanics of complex networks. Rev. Mod. Phys. 74, 47–97 10.1103/RevModPhys.74.47 (doi:10.1103/RevModPhys.74.47)

[48] Dorogovtsev S. N. Mendes J. F. F. 2001 Language as an evolving word web. Proc. R. Soc. Lond. B 268, 2603–2606 10.1098/rspb.2001.1824 (doi:10.1098/rspb.2001.1824) PMC1088922 11749717

[49] Solé R. V. Corominas-Murtra B. Valverde S. Steels L. 2010 Language networks: their structure, function and evolution. Complexity 6, 20–26 10.1002/cplx.20305 (doi:10.1002/cplx.20305)

[50] Michel J. B. & The Google Books Team. 2011 Quantitative analysis of culture using millions of digitized books. Science 331, 176–182 10.1126/science.1199644 (doi:10.1126/science.1199644) PMC3279742 21163965

[51] Ferrer i Cancho R. Solé R. V. 2002 Zipf's law and random texts. Adv. Comp. Syst. 5, 1–6 10.1142/S0219525902000468 (doi:10.1142/S0219525902000468)

[52] Loreto V. Baronchelli A. Mukherjee A. Puglisi A. Tria F. 2011 Statistical physics of language dynamics. J. Stat. Mech. P04006. 10.1088/1742-5468/2011/04/P04006 (doi:10.1088/1742-5468/2011/04/P04006)

[53] Petersen A. M. Tenenbaum J. Havlin S. Stanley H. E. 2012 Statistical laws governing fluctuations in word use from word birth to word death. Sci. Rep. 2, 313 10.1038/srep00313 (doi:10.1038/srep00313) 22423321 PMC3304511

[54] Ferrer i Cancho, R., Solé, R. V 2001 The small-world of human language. Proc. R. Soc. Lond. B 268, 2261–2266 10.1098/rspb.2001.1800 (doi:10.1098/rspb.2001.1800) PMC1088874 11674874

[55] Merton R. K. 1968 The Matthew effect in science. Science 159, 53–63 10.1126/science.159.3810.56 (doi:10.1126/science.159.3810.56) 5634379

[56] de Solla Price D. J 1965 Networks of scientific papers. Science 149, 510–515 10.1126/science.149.3683.510 (doi:10.1126/science.149.3683.510) 14325149

[57] Redner S. 2005 Citation statistics from 110 years of physical review. Phys. Today 58, 49–54 10.1063/1.1996475 (doi:10.1063/1.1996475)

[58] Jeong H. Neda Z. Barabási A.-L. 2003 Measuring preferential attachment for evolving networks. Europhys. Lett. 61, 567–572 10.1209/epl/i2003-00166-9 (doi:10.1209/epl/i2003-00166-9)

[59] Newman M. E. J. 2004 Coauthorship networks and patterns of scientific collaboration. Proc. Natl Acad. Sci. USA 101, 5200–5205 10.1073/pnas.0307545100 (doi:10.1073/pnas.0307545100) 14745042 PMC387296

[60] Petersen A. M. Jung W. S. Yang J. S. Stanley H. E. 2011 Quantitative and empirical demonstration of the Matthew effect in a study of career longevity. Proc. Natl Acad. Sci. USA 108, 18–23 10.1073/pnas.1016733108 (doi:10.1073/pnas.1016733108) 21173276 PMC3017158

[61] Barabási A. L. 2012 The network takeover. Nat. Phys. 8, 14–16 10.1038/nphys2188 (doi:10.1038/nphys2188)

